# Resting-state fMRI correlates of acupuncture in migraine: a coordinate-based meta-analysis

**DOI:** 10.22514/jofph.2026.058

**Published:** 2026-09-12

**Authors:** Guo-Yun Liu, Ling-Hua Li, Zi-Min Cao, Zhe Chen, Zi-Chen Wang, Yi-Kun Yuan, Chao-Qun Yan, Jun Wang

**Affiliations:** ^1^Department of Acupuncture and Moxibustion, Dongzhimen Hospital, Beijing University of Chinese Medicine, 100700 Beijing, China

**Keywords:** Migraine, Acupuncture, Resting-state fMRI, Meta-analysis, SDM-PSI

## Abstract

**Background**: Although acupuncture has shown prophylactic benefits for migraine, its neural mechanisms remain elusive. This study systematically synthesized whole-brain resting-state functional magnetic resonance imaging (fMRI) evidence to characterize changes in spontaneous brain activity after acupuncture treatment in patients with migraine. **Methods**: We systematically searched eight databases for resting-state fMRI studies that used whole-brain analyses to compare patients with migraine before and after acupuncture. Only studies reporting amplitude of low-frequency fluctuation (ALFF) or regional homogeneity (ReHo) were eligible for quantitative meta-analysis. Seed-based d Mapping with Permutation of Subject Images (SDM-PSI) was used to conduct the coordinate-based meta-analysis. Meta-regression analyses explored associations between regional brain changes and clinical outcomes. **Results**: Thirteen studies comprising 19 datasets and 312 patients were included. At the prespecified uncorrected SDM-PSI threshold, acupuncture treatment was associated with increased ALFF in the right middle frontal gyrus and increased ReHo in the left angular gyrus and right superior temporal gyrus, together with decreased ReHo in the right precuneus. Exploratory meta-regression suggested that greater pain reduction was associated with smaller ReHo increases in the right superior temporal gyrus, whereas greater reduction in attack frequency was associated with greater ReHo increases in the left angular gyrus. **Conclusions**: Acupuncture may be associated with altered spontaneous neural activity in regions of the executive control network, default mode network, and multisensory processing regions. These findings provide preliminary neuroimaging evidence for the central correlates of acupuncture in migraine. However, they should be interpreted cautiously given the limited evidence base, methodological heterogeneity, and exploratory nature of the meta-regression analyses. **The PROSPERO Registration**: The review was registered in PROSPERO (CRD420251145641).

## 1. Introduction

Migraine is a neurological disorder characterized by recurrent headache episodes that are commonly accompanied by nausea, vomiting, photophobia, and phonophobia [1]. The Global Burden of Disease Study indicates that migraine imposes a substantial global disability burden and markedly affects patients’ quality of life and social functioning [2, 3, 4]. Epidemiological evidence suggests a global prevalence of approximately 14%–15%, with higher rates among women and the highest prevalence in early to middle adulthood [5, 6, 7]. Although pharmacological options, including triptans and monoclonal antibodies targeting calcitonin gene-related peptide, have expanded therapeutic choices, treatment responses vary among patients. Many individuals continue to experience limited efficacy, adverse effects, overuse of acute medications, or poor tolerance. These challenges underscore the need for effective non-pharmacological approaches [8, 9, 10].

In migraine management, acupuncture is used for both acute symptom relief and prevention of recurrence. Evidence from randomized trials and systematic reviews supports the beneficial role of acupuncture in reducing migraine attack frequency, alleviating pain intensity, and improving patients’ quality of life. These benefits may persist for several months [11, 12, 13, 14, 15]. Nevertheless, the central nervous system mechanisms underlying acupuncture remain incompletely understood. Advances in neuroimaging have provided important tools for investigating these mechanisms [16, 17].

Migraines are increasingly recognized as a brain network disorder. It is accompanied by widespread functional abnormalities in regions involved in pain processing, including the thalamus, periaqueductal gray (PAG), cingulate cortex, and insula. Alterations have also been reported in large-scale intrinsic networks, including the default mode network (DMN), salience network (SN), and executive control network (ECN). These functional imbalances are closely related to migraine chronification and cognitive impairment [18, 19, 20, 21]. Resting-state functional magnetic resonance imaging (rs-fMRI) measures spontaneous low-frequency fluctuations in the blood oxygen level-dependent (BOLD) signal. Metrics such as regional homogeneity (ReHo) and amplitude of low-frequency fluctuation (ALFF/fALFF) characterize local neuronal synchrony and oscillation amplitude. Other neuroimaging modalities, including arterial spin labeling (ASL), positron emission tomography (PET), and single-photon emission computed tomography (SPECT), can further assess cerebral blood flow and metabolism.

Recent neuroimaging studies have shown that acupuncture is accompanied by changes in functional activity and network connectivity across multiple brain regions in patients with migraine. These regions include pain modulation systems, sensory processing areas, and emotion-related regions [22, 23, 24]. However, findings remain heterogeneous because of differences in study design, sample size, acupuncture protocols, and imaging analysis methods. A systematic synthesis of the available evidence is therefore needed.

Coordinate-based meta-analysis (CBMA) provides a quantitative framework for integrating functional neuroimaging findings and identifying brain regions with spatially consistent alterations across studies [25]. By integrating reported peak coordinates across experiments, CBMA enables voxel-level detection of convergent neuroimaging abnormalities and provides a more objective summary than narrative synthesis alone. Seed-based d Mapping with Permutation of Subject Images (SDM-PSI) further supports voxel-wise meta-analytic synthesis based on reported peak coordinates and effect sizes, while accounting for sample size and variability across studies [26].

Therefore, we applied SDM-PSI to systematically synthesize resting-state neuroimaging studies of acupuncture treatment for migraine. The objective was to identify consistent patterns of brain activity changes after acupuncture and to provide preliminary neuroimaging evidence for potential central mechanisms related to its clinical effects.

## 2. Materials and methods

### 2.1 Literature search strategy

This review was reported in accordance with the Preferred Reporting Items for Systematic Reviews and Meta-Analyses (PRISMA) 2020 statement [27]. The PRISMA 2020 checklist is provided as **Supplementary material 1**. We systematically searched PubMed, Web of Science, Embase, the Cochrane Library, China National Knowledge Infrastructure (CNKI), VIP Database (VIP), Wanfang Database, and the Chinese Biomedical Literature Database (CBM) from inception to 08 August 2025. No restrictions were applied regarding language or publication date. The search strategy combined terms for three key concepts using both Medical Subject Headings (MeSH) and free-text keywords: migraine disorders (“Migraine Disorders” [MeSH], “migraine”, “migraine disorders”), acupuncture interventions (“Acupuncture” [MeSH], “Acupuncture Therapy” [MeSH], “acupuncture”, “electroacupuncture”, “acupuncture needle”), and neuroimaging techniques (“Neuroimaging” [MeSH], “Magnetic Resonance Imaging” [MeSH], “fMRI”, “rs-fMRI”, “PET”, “SPECT”, “ASL”, “ALFF”, “fALFF”, “ReHo”). These three components were combined using the Boolean operator “AND” to retrieve relevant studies. The search strategy was adapted for each database, and detailed strategies for all databases are presented in **Supplementary Table 1**. We also screened trial registries and the reference lists of eligible articles and related reviews to identify additional studies. Study selection was performed independently by two reviewers (GYL and LHL). Discrepancies were resolved through consultation, and a third senior reviewer (ZMC) arbitrated when necessary. The review protocol was registered in the International Prospective Register of Systematic Reviews (PROSPERO/CRD420251145641). The overall study selection process is illustrated in Fig. 1.

**Fig. 1.** PRISMA flow diagram of the study identification and selection process. n, number.

### 2.2 Selection criteria

Eligible studies met the following criteria: (1) participants were diagnosed with migraine according to the International Classification of Headache Disorders (ICHD) criteria; (2) the intervention involved a standardized acupuncture protocol, with details of acupoints, needling technique, treatment duration, and treatment frequency reported, and resting-state brain function was assessed both before and after the treatment course; (3) the study included pre- and post-intervention measurements, including but not limited to single-group pre–post designs, non-randomized controlled trials, and randomized controlled trials; (4) resting-state neuroimaging techniques were used, including but not limited to ALFF, fALFF, ReHo, cerebral blood flow (CBF), ASL, PET, or SPECT; and (5) whole-brain analyses were performed, with three-dimensional peak coordinates reported in Montreal Neurological Institute (MNI) or Talairach space together with associated statistical values.

Studies were excluded if: (1) acupuncture groups included fewer than seven participants [28]; (2) region-of-interest (ROI)-based analyses were used, or whole-brain peak coordinates and associated statistics could not be extracted; (3) the publication was a case report, narrative review, meta-analysis, or animal study; or (4) data were duplicated across multiple publications.

### 2.3 Data extraction

After removal of ineligible records, two reviewers (GYL and LHL) independently extracted data from the eligible studies. Disagreements were resolved by consensus. Extracted demographic variables included sample size, mean age, and sex distribution. Clinical variables included migraine subtype, disease duration, Visual Analogue Scale (VAS) scores, and attack frequency. Additional extracted information included diagnostic criteria, intervention protocols, imaging methodology, peak coordinates, and *t*-values or other comparable statistical parameters. Unreported data were recorded as not available (NA).

### 2.4 Quality assessment

In the absence of established guidelines for evaluating fMRI study quality, we developed a 12-item checklist (**Supplementary Table 2**) based on criteria used in previous neuroimaging meta-analyses [29, 30]. Each item was assigned 1, 0.5, or 0 points according to whether the criterion was fully met, partially met, or not met. A score of 0.5 was assigned when relevant information was reported but incomplete, or when the criterion was only partially satisfied. The purpose of this assessment was to descriptively summarize reporting completeness and methodological characteristics of the included studies. It was not intended to exclude studies, weight the meta-analysis, evaluate clinical efficacy, or determine the certainty of clinical evidence [31]. The checklist covered three domains: sample characteristics, image acquisition and analysis, and results and conclusions. Based on the total score, studies were grouped into three quality levels: high (>10), moderate (6–10), and low (<6). These thresholds were defined a priori and applied uniformly to all neuroimaging studies. Quality ratings were performed independently by two reviewers (GYL and LHL), and any remaining disagreements were resolved through discussion with a senior reviewer (ZMC). The checklist items and item-level scores for each included study are summarized in **Supplementary Tables 2,3**.

### 2.5 Data analysis

#### 2.5.1 Voxel-based meta-analysis

SDM-PSI is a voxel-wise meta-analytic approach that reconstructs effect-size maps from reported peak coordinates and corresponding statistics, while weighting results by sample size and other study-level factors. We conducted voxel-wise meta-analyses using the SDM-PSI graphical user interface (version 6.23; SDM Project, London, UK) to examine resting-state brain changes after acupuncture in patients with migraine [32]. The analytical procedure used in the present study is shown in Fig. 2. For all statistically significant clusters, peak coordinates and associated effect sizes were extracted. For studies reporting only *z* values or *p* values, *t* values were derived using the SDM online transformation tool. Talairach coordinates were converted to MNI space before further processing. Based on these data, SDM-PSI generated effect-size maps in standard MNI space and incorporated anisotropic Gaussian smoothing at the study level. The mean map was then derived using a random-effects meta-analysis that incorporated sample size, within-study variance, and between-study heterogeneity. Default SDM parameters were applied (full width at half maximum = 20 mm; uncorrected voxel-level *p* < 0.005; peak height Z > 1; cluster extent >10 voxels), as these settings are considered to provide an appropriate balance between false-positive and false-negative findings.

**Fig. 2.** SDM-PSI meta-analysis pipeline for ALFF and ReHo changes after acupuncture. Red upward arrows (↑) indicate increased ALFF/ReHo values after acupuncture, whereas blue downward arrows (↓) indicate decreased ALFF/ReHo values. ALFF, amplitude of low-frequency fluctuation; FWE, family-wise error; MNI, Montreal Neurological Institute; ReHo, regional homogeneity; SDM-PSI, seed-based d mapping with permutation of subject images; TFCE, threshold-free cluster enhancement.

#### 2.5.2 Jackknife sensitivity analysis

A jackknife procedure was performed to assess the stability of the meta-analytic results. Each study was systematically excluded in turn, and the outcomes were recalculated using the remaining dataset to determine whether the results were disproportionately influenced by any individual study [33]. Brain regions that remained significant across all iterations were considered stable.

#### 2.5.3 Heterogeneity and publication bias

Between-study heterogeneity was assessed using *I*^2^ statistics derived from the random-effects model for each significant cluster identified in the primary meta-analysis. Brain regions with *I*^2^ ≥ 50% were considered to exhibit substantial heterogeneity. Heterogeneous clusters were identified using the standard SDM kernel and thresholds (FWHM = 20 mm, uncorrected *p* = 0.005, peak height Z > 1, cluster extent = 10 voxels) [34]. Potential publication bias was examined using Egger’s test [35] and was visually inspected with funnel plots based on statistically significant peaks [36].

#### 2.5.4 Meta-regression analysis

Voxel-wise meta-regression was conducted to examine associations between clinical variables and imaging findings. The assessed variables included age, disease duration, sex ratio, change in VAS score after acupuncture (ΔVAS = pre-treatment − post-treatment), and change in headache frequency (Δfrequency = pre-treatment − post-treatment). The analysis was restricted to brain regions that were significant in the main meta-analysis. Following previous SDM-PSI studies, we adopted more stringent exploratory thresholds (voxel-wise *p* < 0.0005 and cluster extent ≥10 voxels) to reduce the risk of false-positive associations [37].

## 3. Results

### 3.1 Included studies and sample characteristics

The systematic literature search identified 789 records. After duplicate removal, 356 records remained for title and abstract screening. Following screening, 64 reports were assessed for full-text eligibility, and 13 studies ultimately met the inclusion criteria and were included in the review (Fig. 1) [38, 39, 40, 41, 42, 43, 44, 45, 46, 47, 48, 49, 50]. Although the initial search identified multiple neuroimaging modalities, including ALFF, ReHo, CBF, ASL, PET, and SPECT, only studies that used ALFF or ReHo metrics, conducted whole-brain analyses before and after acupuncture, and reported three-dimensional stereotactic coordinates were eligible for quantitative meta-analysis.

A total of 19 independent datasets were extracted from the 13 studies, encompassing 312 patients with migraine. The ALFF meta-analysis included 7 datasets (n = 249), whereas the ReHo meta-analysis included 12 datasets (n = 198). Quality assessment was conducted using the 12-item checklist, and all studies scored ≥10 points (Fig. 3, **Supplementary Tables 2,3**). Among them, 11 studies were rated as high quality (>10) and 2 as moderate quality (6–10). These ratings indicate generally good reporting completeness, because the checklist assessed reporting completeness rather than methodological quality or risk of bias. Study characteristics are presented in Table 1 (Ref. [38, 39, 40, 41, 42, 43, 44, 45, 46, 47, 48, 49, 50]), and neuroimaging parameters are presented in Table 2 (Ref. [38, 39, 40, 41, 42, 43, 44, 45, 46, 47, 48, 49, 50]).

**Fig. 3.** Item-level quality assessment of the 13 included studies based on the 12-item reporting checklist.Each row represents one study, and each column represents one checklist criterion. C1–C12 correspond to the criteria listed in **Supplementary Table 2**. The criteria were grouped into three domains: sample characteristics (C1–C4), image acquisition and analysis (C5–C10), and results and conclusions (C11–C12). Scores of 0, 0.5, and 1 indicate that the criterion was not met, partially met, or fully met, respectively. The rightmost column shows the total checklist score for each study.

**Table 1.** Demographic and clinical characteristics of the included studies.

Article	Type	Sample size (M/C)	Female (M/C)	Age, yr (M/C)	Mean duration, yr	Attack frequency/mon	Diagnostic criteria	Acupuncture protocol	Regimen	Outcomes	Comparator
Gao *et al*. [38] 2025	MWoA	16/16	NA/NA	37.5/34.94	7.52	4.18	ICHD-3	GB20, GB8, GV20	3 times/wk, 12 sessions	VAS, HIT-6, MSQ	Sham acupuncture
Han *et al*. [39] 2017	MWoA	10/10	8/NA	31.7/NA	NA	NA	ICHD-2	GB41	Immediate acupuncture	NA	Healthy control
Ning *et al*. [40] 2020	MWoA	19/18	16/14	28.23/27.16	6.47	NA	ICHD-2	GB41	Immediate acupuncture	NA	Healthy control
Jia *et al*. [41] 2021	MWoA	15/NA	10/NA	39.3/NA	NA	5.5	ICHD-3	GB44, ST45, BL67, LR1	3 times/wk, 12 sessions	VAS, MSQ, SAS, SDS	NA
Wang *et al*. [42] 2021	MM	9/10	9/NA	NA/NA	NA	NA	ICHD-3	GB20, GB8, LR3	2–3 times/wk, 3 mon, 27 ± 6 sessions	VAS	Healthy control
								GB20, GB8, PC6		VAS	
Zhang *et al*. [43] 2024	MWoA	28/NA	24/NA	37.82/NA	16.42	NA	ICHD-3	GV20, GV24, GB13, GB8, GB20	Immediate acupuncture	VAS, HIT-6, BAI, BDI	NA
Zhao *et al*. [44] 2014	MWoA	20/20	14/12	32.9/37.25	8.55	10.40	ICHD-2	SJ5, GB20, GB34, GB40	4 times/wk, 8 wk, 32 sessions	VAS, HIT-6, attack days, attack frequency	Inactive acupoints
Zhang *et al*. [45] 2021	MM	24/20	24/NA	33.04/35.30	NA	3.29	ICHD-3 beta	GB20, GB8, PC6, SP6, LR3	2–3 times/wk, 3 mon, 27 ± 6 sessions	SAS, SDS, VAS, attack frequency	Sham acupuncture
Yang *et al*. [46] 2024	MWoA	50/50	50/50	28.36/27.60	1.20	1.46	ICHD-3	GV20, GB8, GB5, ST8	2 times/wk, 4 wk, 8 sessions	MIDAS, SF-MPQ	Healthy control
Xie *et al*. [47] 2025	MWoA	30/26	22/25	33.67/33.42	5.94	4.17	ICHD-3	GV20, GB20, GB8	3 times/wk, 4 wk, 12 sessions	VAS, HIT-6, MSQ, attack frequency, attack days	Sham acupuncture
Li *et al*. [48] 2017	MWoA	35/11	27/9	21.3/21.18	5.53	6.3	ICHD-2	GB34, GB40, SJ5, GB33, GB42, SJ8, ST36, ST42, LI6	5 times/wk, 4 wk, 20 sessions	VAS, SAS, SDS, attack frequency	Sham acupuncture; Healthy control; Waiting list
Liu *et al*. [49] 2021	MWoA	37/15	31/13	37.97/34.88	16.19	5.16	ICHD-3	GV20, EX-HN5, GB20, GB8, GB5, GB15, LI4, LR3	2 times/wk, 6 wk, 12 sessions	VAS, MSQ, SAS, SDS, attack days	Healthy control
Li *et al*. [50] 2023	MWoA	10/9	8/5	37.4/33.0	10	10	ICHD-3	GB20, LR3, EX-HN5, GV20, EX-HN1	Two courses, 5 days each, 1-day interval between courses	VAS, PSQI, GAD-7, MSQ, attack frequency	Sham acupuncture; Healthy control; Blank control

*M, migraine group; C, control group; NA, not available; BAI, Beck Anxiety Inventory; BDI, Beck Depression Inventory; GAD-7, Generalized Anxiety Disorder-7; HIT-6, Headache Impact Test-6; ICHD, International Classification of Headache Disorders; MIDAS, Migraine Disability Assessment; MM, menstrual migraine; MSQ, Migraine-Specific Quality of Life Questionnaire; MWoA, migraine without aura; PSQI, Pittsburgh Sleep Quality Index; SAS, Self-Rating Anxiety Scale; SDS, Self-Rating Depression Scale; SF-MPQ, Short-Form McGill Pain Questionnaire; VAS, Visual Analogue Scale. Acupoint codes: BL67, Zhiyin; EX-HN1, Sishencong; EX-HN5, Taiyang; GB13, Benshen; GB15, Toulinqi; GB20, Fengchi; GB33, Xiyangguan; GB34, Yanglingquan; GB40, Qiuxu; GB41, Zulinqi; GB42, Diwuhui; GB44, Zuqiaoyin; GB5, Xuanlu; GB8, Shuaigu; GV20, Baihui; GV24, Shenting; LI4, Hegu; LI6, Pianli; LR1, Dadun; LR3, Taichong; PC6, Neiguan; SJ5, Waiguan; SJ8, Sanyangluo; SP6, Sanyinjiao; ST36, Zusanli; ST42, Chongyang; ST45, Lidui; ST8, Touwei.*

**Table 2.** Technical details of the included studies.

Study	Gray matter	Coil channel	Software	FWHM	Space	Threshold	Increase	Decrease	Quality score
Gao *et al*. [38] 2025	ReHo	NA	SPM 8, DPARSF	6	Talairach	*p *< 0.05, GRF corrected	YES	YES	12
Han *et al*. [39] 2017	ReHo	NA	SPM 8, REST, DPARSF	NA	MNI	*p *< 0.05, Monte Carlo corrected, cluster size >604 mm²	NO	YES	10
Ning *et al*. [40] 2020	ALFF	NA	DPARSF, REST	4	MNI	*p *< 0.05, Monte Carlo corrected, cluster size ≥70 mm²	YES	YES	11.5
Jia *et al*. [41] 2021	ReHo	NA	DPARSF	NA	MNI	*p *< 0.05, GRF corrected	NO	YES	11.5
Wang *et al*. [42] 2021	ReHo	NA	SPM 12	NA	Talairach	*p *< 0.05, GRF corrected	YES	YES	10
Zhang *et al*. [43] 2024	mReHo	20	SPM 8, DPARSF	4	MNI	Voxel: *p *< 0.001, uncorrected; Cluster: *p *< 0.05, FWE corrected	YES	YES	12
Zhao *et al*. [44] 2014	ReHo	8	SPM 5, REST	4	Talairach	*p *< 0.05, FDR corrected, cluster size ≥20 voxels	YES	YES	12
Zhang *et al*. [45] 2021	ALFF	8	SPM 12, DPABI	NA	Talairach	*p *< 0.05, FDR corrected	YES	YES	11.5
	ReHo						YES	YES	
Yang *et al*. [46] 2024	ALFF	32	SPM 12, REST	6	MNI	*p *< 0.001, FDR corrected, cluster size >20 voxels	NO	YES	12
Xie *et al*. [47] 2025	ALFF	8	SPM 8, DPABI	6	MNI	Voxel: *p *< 0.001, uncorrected; Cluster: *p *< 0.05, GRF corrected	YES	YES	12
	fALFF						YES	NO	
	ReHo						YES	YES	
Li *et al*. [48] 2017	ALFF	8	SPM 12, DPARSF, REST	6	MNI	Voxel: *p *< 0.001, uncorrected; Cluster: *p *< 0.05, FWE corrected	YES	YES	12
Liu *et al*. [49] 2021	ReHo	12	SPM 12	6	MNI	*p *< 0.05, FWE corrected	YES	NO	12
	ReHo						YES	NO	
Li *et al*. [50] 2023	ALFF	NA	SPM12, DPABI	NA	MNI	*p *< 0.001, uncorrected, cluster size ≥5 voxels	YES	YES	10.5
	ReHo						NO	YES	

*All studies used a 3.0 T MRI scanner. ALFF, amplitude of low-frequency fluctuation; fALFF, fractional ALFF; FDR, false discovery rate; FWE, family-wise error; FWHM, full width at half maximum; GRF, Gaussian random field; MNI, Montreal Neurological Institute; mReHo, mean regional homogeneity; NA, not available; ReHo, regional homogeneity; REST, Resting-State fMRI Data Analysis Toolkit; SPM, Statistical Parametric Mapping; DPARSF, Data Processing Assistant for Resting-State fMRI; DPABI, Data Processing & Analysis for Brain Imaging.*

### 3.2 Meta-analysis of ALFF

Voxel-level meta-analysis based on SDM-PSI showed that, relative to the pre-acupuncture baseline, patients with migraine exhibited significantly increased ALFF in the right middle frontal gyrus (Brodmann area (BA) 9) after acupuncture treatment (MNI coordinates: 30, 38, 34; SDM-Z = 3.540; *p* = 0.0002; cluster volume = 183 voxels) (Fig. 4A, Table 3). No significant ALFF decreases were detected in any brain region. After family-wise error (FWE) correction, these differences were no longer statistically significant.

**Table 3.** Brain regions showing significant changes in ALFF and ReHo after acupuncture treatment.

Measure	Contrast	Direction	Brain Region (BA)	MNI (x, y, z)	SDM-Z	*p*-value	Cluster size (voxels)	*I*²	Egger’s test (*p*-value)
ALFF	Post > Pre	Increase	Right Middle Frontal Gyrus (BA 9)	30, 38, 34	3.540	0.00020	183	7.362	0.810
ReHo	Post > Pre	Increase	Left Angular Gyrus (BA 39)	−52, −62, 30	3.230	0.00062	39	11.367	0.887
ReHo	Post > Pre	Increase	Right Superior Temporal Gyrus (BA 21)	54, 0, −12	2.798	0.00257	20	13.631	0.780
ReHo	Post < Pre	Decrease	Right Precuneus	2, −52, 48	−2.968	0.00150	62	3.126	0.839
ReHo	Post < Pre	Decrease	Right Precuneus	6, −44, 46	−2.791	0.00262	10	16.580	0.733

*ALFF, amplitude of low-frequency fluctuation; ReHo, regional homogeneity; BA, Brodmann area; MNI, Montreal Neurological Institute coordinates; SDM-Z, seed-based d mapping Z-score; I^2^, heterogeneity statistic; Post, post-treatment; Pre, pre-treatment.*

The ALFF finding was relatively stable in sensitivity analyses and showed no clear evidence of publication bias. Jackknife sensitivity analysis indicated that the result was reproducible across most study combinations (**Supplementary Table 4**, Ref. [38, 39, 40, 41, 42, 43, 44, 45, 46, 47, 48, 49, 50]). Although the inclusion of fewer than 10 studies limited the statistical power of the publication bias test, heterogeneity analysis showed a low *I*^2^ value, and Egger’s test did not indicate significant publication bias (*p* > 0.05) (Table 3). The funnel plot distribution was largely symmetrical (**Supplementary Fig. 1**), providing no clear evidence of major publication bias in the ALFF meta-analysis.

### 3.3 Meta-analysis of ReHo

Voxel-level meta-analysis based on SDM-PSI revealed significant ReHo changes across multiple brain regions in patients with migraine after acupuncture treatment relative to baseline (Fig. 4B, Table 3).

**Fig. 4.** Brain regions with significantly altered ALFF and ReHo after acupuncture treatment in patients with migraine. (A) Brain regions showing significantly increased ALFF after acupuncture compared with baseline. (B) Brain regions showing significantly increased and decreased ReHo after acupuncture compared with baseline. Warm colors indicate increased ALFF/ReHo, whereas cold colors indicate decreased ReHo. The numbers beneath the images indicate z-coordinates of the slices in MNI space. L, left; R, right.

Brain regions showing increased ReHo included the left angular gyrus (BA 39; MNI = −52, −62, 30; SDM-Z = 3.230; *p* = 0.0006; cluster volume = 39 voxels) and the right superior temporal gyrus (BA 21; MNI = 54, 0, −12; SDM-Z = 2.798; *p* = 0.0026; cluster volume = 20 voxels).

Decreased ReHo was observed in the right precuneus at two peak locations (Peak 1: MNI = 2, −52, 48; SDM-Z = −2.968; *p *= 0.0015; cluster volume = 62 voxels; Peak 2: MNI = 6, −44, 46; SDM-Z = −2.791; *p* = 0.0026; cluster volume = 10 voxels). After FWE correction, these differences were no longer statistically significant.

Sensitivity analyses and publication bias assessments provided supportive evidence for the stability of these findings. Jackknife sensitivity analysis indicated that the ReHo findings were reproducible across most study combinations (**Supplementary Table 4**). Heterogeneity analysis showed low *I*^2^ values, and Egger’s test did not suggest significant publication bias (*p* > 0.05) (Table 3). The funnel plot distribution was largely symmetrical (**Supplementary Fig. 1**), suggesting no clear evidence of major publication bias in the ReHo analysis.

### 3.4 Multimodal analysis

The multimodal analysis did not identify any brain regions with overlapping ALFF and ReHo changes after acupuncture. Specifically, no regions showed simultaneous increases or decreases in both measures, and no regions showed opposite directions of change between ALFF and ReHo.

### 3.5 Meta-regression analysis

Meta-regression analyses were performed to examine potential confounding factors and associations between clinical improvement and brain functional changes. Using the more stringent exploratory thresholds (*p* < 0.0005, cluster size ≥10 voxels), age, sex ratio, disease duration, and baseline clinical measures showed no significant effects on ALFF or ReHo.

Exploratory analyses using an uncorrected threshold (*p *< 0.05, cluster size ≥10 voxels) revealed significant correlations between clinical improvement and regional brain activity (Fig. 5). Reduction in VAS score (ΔVAS = pre-treatment − post-treatment) was negatively correlated with ReHo in the right superior temporal gyrus (BA 21; MNI: 62, −6, −4; SDM-Z = −2.468; *p* = 0.007; 406 voxels), suggesting that greater pain relief was associated with a smaller increase in local neural synchrony. Reduction in attack frequency (Δfrequency = pre-treatment − post-treatment) was positively correlated with ReHo in the left angular gyrus (BA 39; MNI: −54, −60, 30; SDM-Z = 2.617; *p* = 0.004; 115 voxels), indicating that greater reduction in attack frequency was associated with greater local neural synchrony.

**Fig. 5.** Exploratory meta-regression results (uncorrected *p* < 0.05, cluster size ≥10 voxels). (a) ΔVAS was negatively associated with ReHo in the right superior temporal gyrus. (b) Δfrequency was positively associated with ReHo in the left angular gyrus. L, left; R, right; SDM, seed-based d mapping; ReHo, regional homogeneity; VAS, Visual Analogue Scale; ΔVAS, pre-treatment minus post-treatment VAS score; Δfrequency, pre-treatment minus post-treatment migraine attack frequency.

## 4. Discussion

We conducted a coordinate-based meta-analysis using SDM-PSI to synthesize resting-state neuroimaging evidence on acupuncture treatment for migraine. Acupuncture was associated with alterations in spontaneous neural activity across several cortical regions, including increased ALFF in the middle frontal gyrus, increased ReHo in the angular and superior temporal gyri, and decreased ReHo in the precuneus. Exploratory meta-regression further suggested that improvement in VAS score was negatively associated with ReHo changes in the right superior temporal gyrus, whereas reduction in attack frequency was positively associated with ReHo changes in the left angular gyrus. Jackknife sensitivity and heterogeneity analyses were performed to examine the stability and variability of these findings.

We observed increased ALFF in the right middle frontal gyrus after acupuncture treatment compared with baseline. The middle frontal gyrus is a major component of the dorsolateral prefrontal cortex (dlPFC) and an important node of the executive control network (ECN) [51]. It contributes to cognitive pain modulation, attentional control, and descending pain inhibition [52, 53]. Previous studies have reported reduced prefrontal functional connectivity in patients with migraine, and these reductions are negatively correlated with pain intensity during attacks [54, 55]. Structural imaging studies have also identified decreased gray matter volume and gyrification in the middle frontal gyrus in patients with migraine without aura [56, 57]. The dlPFC can engage descending pain-modulatory pathways through projections to brainstem structures such as the PAG [58, 59]. Therefore, elevated ALFF in the middle frontal gyrus may reflect compensatory or treatment-related changes in ECN-related regions involved in top-down prefrontal modulation of pain processing.

In this study, acupuncture was associated with bidirectional ReHo changes. The angular gyrus, located at the junction of the occipital, temporal, and parietal lobes, is a key hub of the DMN and supports cross-modal integration of sensory, cognitive, and affective information. In migraine, abnormal angular gyrus connectivity has been linked to disturbances in attention, working memory, and executive control, and its dysfunction has been implicated in migraine chronification [60, 61]. As an interface region linking the DMN and ECN, the angular gyrus may contribute to coordination among large-scale brain networks [49]. The observed increase in left angular gyrus ReHo may indicate altered local neural synchrony after acupuncture. This change may be related to multisensory integration and cognitive control processes involved in pain modulation.

The precuneus is a highly connected hub within the DMN and is involved in self-referential processing, internal attentional regulation, and integration of pain perception [62]. Previous studies have reported increased DMN functional connectivity and ReHo values in the precuneus/posterior cingulate cortex in patients with migraine without aura [63]. These findings suggest that hyperactivation in this region may contribute to excessive pain-related attention and aberrant self-referential processing. In addition, reduced functional connectivity between the right frontoparietal network (FPN) and the precuneus has been associated with decreased migraine intensity. This pattern has been interpreted as a compensatory mechanism that may alleviate pain experience by reducing attentional focus on pain [64]. In the present study, decreased ReHo in the precuneus after acupuncture may reflect a shift from abnormal hypersynchrony toward a more normalized functional state. Such a shift could reduce excessive pain-related attention and facilitate cognitive control of pain through descending modulation [44]. However, this interpretation remains indirect because ALFF and ReHo do not directly measure connectivity between regions.

The superior temporal gyrus is a key part of the auditory association cortex and is involved in sound perception, emotion regulation, and audiovisual integration [65, 66]. Patients with migraine often show heightened sensitivity to visual and auditory stimuli. Neuroimaging studies have linked this hypersensitivity to dysfunction in multisensory integration regions, including the superior temporal gyrus [67]. Other studies have reported reduced effective connectivity from the amygdala to the bilateral superior temporal gyri in patients with migraine, suggesting weakened coupling between emotional and sensory processing that may contribute to auditory hypersensitivity and temporal processing deficits [68]. Previous work has reported enhanced local neural synchrony in the superior temporal gyrus in patients with migraine, which has been interpreted as a compensatory response to increased demands for multisensory and affective integration [67]. In the present study, higher ReHo in the superior temporal gyrus after acupuncture may indicate altered activity in regions implicated in auditory and affective processing. This pattern may be linked to migraine-related sensory or affective processing and multisensory integration.

Exploratory meta-regression using an uncorrected threshold (*p* < 0.05) showed that VAS improvement was negatively correlated with ReHo changes in the right superior temporal gyrus. This finding may suggest that pain relief is accompanied by functional changes in sensory processing regions, although the direction and biological significance of this association require further investigation. Reduction in attack frequency was positively correlated with ReHo changes in the left angular gyrus, which may reflect local functional alterations involved in longer-term symptom regulation. These opposing associations suggest that improvements in pain intensity and attack frequency may correspond to partly distinct patterns of brain activity change after acupuncture. Because these findings were derived using an uncorrected exploratory threshold, they should be regarded as preliminary and require replication in larger cohorts.

The absence of spatial overlap between ALFF and ReHo results indicates that the two measures did not identify the same regions of change. This lack of overlap may partly reflect the limited number of included studies, insufficient statistical power, and methodological heterogeneity. It may also indicate that ALFF and ReHo capture different aspects of local brain activity after acupuncture; therefore, divergence between these measures does not necessarily imply inconsistency. Because no region was identified by both indices, the regional findings should be interpreted as complementary patterns rather than mutually corroborated evidence. Jackknife sensitivity analysis and heterogeneity assessment supported the overall stability of the main regional findings. The persistence of these findings in jackknife analyses, together with the absence of substantial statistical heterogeneity, supports the robustness of the main regional effects and suggests that they were not primarily influenced by any single dataset or marked between-study variability. Nevertheless, the limited number of eligible studies and differences in study design require cautious interpretation. These regional findings should be confirmed in larger, independent datasets before being considered robust.

Taken together, these findings suggest that acupuncture for migraine may be accompanied by changes in neural activity across multiple cortical regions involved in pain processing (Fig. 6). We emphasize that ALFF and ReHo are local indices and cannot directly assess connectivity between regions. The observed alterations involve regions within broad brain networks and may provide preliminary clues for identifying candidate targets in future mechanistic studies. Future research should incorporate functional and effective connectivity analyses to examine how these regions interact with broader pain-modulation networks during brain responses to acupuncture in migraine.

**Fig. 6.** Proposed neural correlates of acupuncture in migraine. Upward arrows (↑) indicate increased ALFF/ReHo values after acupuncture, whereas downward arrows (↓) indicate decreased ALFF/ReHo values. DMN, default mode network; ECN, executive control network; SN, salience network; ReHo, regional homogeneity; ALFF, amplitude of low-frequency fluctuation; VAS, Visual Analogue Scale.

This study has several limitations. First, coordinate-based meta-analysis relies on published peak coordinates rather than full statistical maps, which may introduce reporting bias. Second, the number of included studies was limited because many investigations used region-of-interest analyses or reported only descriptive findings without whole-brain coordinates. This limitation constrained the statistical power of the meta-analysis and reduced the robustness of the results. Third, the small number of eligible studies precluded subgroup analyses based on migraine subtype (with or without aura), acupuncture protocol (acupoint selection and needle retention duration), or treatment duration, all of which may differentially influence brain functional changes. Fourth, methodological heterogeneity in scanning parameters, preprocessing pipelines, statistical thresholds, and multiple-comparison correction strategies may have affected the precision and comparability of the findings, including the spatial distribution of reported peak coordinates. Finally, the meta-regression analyses used an exploratory threshold (uncorrected *p *< 0.05) to maximize sensitivity; therefore, these results should be considered preliminary and require confirmation in independent samples. Because this review focused on neuroimaging outcomes, treatment safety was neither a prespecified outcome nor consistently reported across studies, and was therefore not evaluated. Future neuroimaging studies should systematically report tolerability and adverse events to place mechanistic findings in the context of treatment safety.

This meta-analysis provides a preliminary synthesis of neuroimaging evidence related to acupuncture treatment for migraine. The primary ALFF and ReHo findings did not survive family-wise error correction, likely because of the small number of available datasets, and should therefore be regarded as exploratory. In addition, heterogeneity in study designs indicates that the pooled findings should be interpreted as brain activity changes accompanying treatment rather than as effects causally attributable to acupuncture.

## 5. Conclusions

This coordinate-based meta-analysis of resting-state functional neuroimaging studies suggests that acupuncture for migraine is associated with altered spontaneous brain activity in several regions linked to cognitive control, default mode processing, and multisensory integration, including the right middle frontal gyrus, left angular gyrus, right precuneus, and right superior temporal gyrus. These alterations in hub regions within large-scale brain networks may be related to central processes underlying acupuncture treatment for migraine. Further prospective studies using standardized acupuncture and imaging protocols with complete whole-brain reporting are needed to test the reproducibility of these patterns and to clarify whether they have value as candidate targets or imaging markers of treatment response.

## Supplementary Material

Supplementary material associated with this article can be found, in the online version, at https://files.jofph.com/files/article/2098292849336303616/attachment/Supplementary%20material.zip.




