# Supplementary material for: Resting-state fMRI correlates of acupuncture in migraine: a coordinate-based meta-analysis
Source: J Oral Facial Pain Headache. 2026 Sep 12;40(5):24–38. doi: 10.22514/jofph.2026.058 (PMC13678556; doi:10.22514/jofph.2026.058)
Supplement: Supplementary file 2 [file Supplementary-material-2.docx]

Supplementary material

Supplementary Table 1. Search strategies.

| # | Searches |
| --- | --- |
| **PubMed** | |
| 1 | Migraine Disorders[MeSH] |
| 2 | migraine OR migraine disorders[Title/Abstract] |
| 3 | OR/1–2 |
| 4 | Acupuncture[MeSH] |
| 5 | Acupuncture Therapy[MeSH] |
| 6 | acupuncture OR electroacupuncture OR acupuncture needle[Title/Abstract] |
| 7 | OR/4–6 |
| 8 | Neuroimaging[MeSH] |
| 9 | Magnetic Resonance Imaging[MeSH] |
| 10 | neuroimaging OR magnetic resonance imaging OR functional magnetic resonance imaging OR fMRI OR resting-state fMRI OR rs-fMRI[Title/Abstract] |
| 11 | PET OR positron emission tomography OR SPECT OR single photon emission computed tomography[Title/Abstract] |
| 12 | ASL OR arterial spin labeling OR cerebral blood flow OR CBF[Title/Abstract] |
| 13 | Amplitude of Low Frequency Fluctuation OR ALFF OR fractional ALFF OR fALFF OR regional homogeneity OR ReHo[Title/Abstract] |
| 14 | OR/8–13 |
| 15 | 3 AND 7 AND 14 |
| **Cochrane Library** | |
| 1 | MeSH descriptor: [Migraine Disorders] explode all trees |
| 2 | MeSH descriptor: [Migraine without Aura] explode all trees |
| 3 | MeSH descriptor: [Migraine with Aura] explode all trees |
| 4 | (migraine OR migraine disorders OR Migraine without Aura OR Migraine with Aura):ti,ab,kw |
| 5 | MeSH descriptor: [Acupuncture] explode all trees |
| 6 | MeSH descriptor: [Acupuncture Therapy] explode all trees |
| 7 | (acupuncture OR acupuncture therapy OR electroacupuncture OR acupuncture needle):ti,ab,kw |
| 8 | MeSH descriptor: [Neuroimaging] explode all trees |
| 9 | MeSH descriptor: [Magnetic Resonance Imaging] explode all trees |
| 10 | (neuroimaging OR magnetic resonance imaging OR functional magnetic resonance imaging OR fMRI OR resting-state fMRI OR rs-fMRI OR PET OR positron emission tomography OR SPECT OR single photon emission computed tomography OR ASL OR arterial spin labeling OR cerebral blood flow OR CBF OR Amplitude of Low Frequency Fluctuation OR ALFF OR fractional ALFF OR fALFF OR regional homogeneity OR ReHo):ti,ab,kw |
| 11 | 1 OR 2 OR 3 OR 4 |
| 12 | 5 OR 6 OR 7 |
| 13 | 8 OR 9 OR 10 |
| 14 | 11 AND 12 AND 13 |
| **Embase** | |
| 1 | migraine:ab,ti OR ‘migraine disorders’:ab,ti OR ‘migraine without aura’:ab,ti OR ‘migraine with aura’:ab,ti |
| 2 | acupuncture:ab,ti OR ‘acupuncture therapy’:ab,ti OR electroacupuncture:ab,ti OR ‘acupuncture needle’:ab,ti |
| 3 | neuroimaging:ab,ti OR ‘magnetic resonance imaging’:ab,ti OR ‘functional magnetic resonance imaging’:ab,ti OR fmri:ab,ti OR ‘resting-state fmri’:ab,ti OR ‘rs fmri’:ab,ti OR pet:ab,ti OR ‘positron emission tomography’:ab,ti OR spect:ab,ti OR ‘single photon emission computed tomography’:ab,ti OR asl:ab,ti OR ‘arterial spin labeling’:ab,ti OR ‘cerebral blood flow’:ab,ti OR cbf:ab,ti OR ‘amplitude of low frequency fluctuation’:ab,ti OR alff:ab,ti OR ‘fractional alff’:ab,ti OR falff:ab,ti OR ‘regional homogeneity’:ab,ti OR reho:ab,ti |
| 4 | 1 AND 2 AND 3 |
| **Web of Science** | |
| 1 | TS=(Migraine Disorders OR Migraine without Aura OR Migraine with Aura) OR AB=(migraine OR migraine disorders OR Migraine without Aura OR Migraine with Aura) |
| 2 | TS=(Acupuncture OR Acupuncture Therapy) OR AB=(acupuncture OR electroacupuncture OR acupuncture needle) |
| 3 | TS=(Neuroimaging OR Magnetic Resonance Imaging) OR AB=(neuroimaging OR magnetic resonance imaging OR functional magnetic resonance imaging OR fMRI OR resting-state fMRI OR rs-fMRI OR PET OR positron emission tomography OR SPECT OR single photon emission computed tomography OR ASL OR arterial spin labeling OR cerebral blood flow OR CBF OR Amplitude of Low Frequency Fluctuation OR ALFF OR fractional ALFF OR fALFF OR regional homogeneity OR ReHo) |
| 4 | 1 AND 2 AND 3 |
| **CNKI** | |
| 1 | Title/Keyword/Abstract = Chinese-English expanded search (needle) |
| 2 | Title/Keyword/Abstract = Chinese-English expanded search (acupuncture) |
| 3 | Title/Keyword/Abstract = Chinese-English expanded search (acupuncture needling) |
| 4 | Title/Keyword/Abstract = Chinese-English expanded search (electroacupuncture) |
| 5 | Title/Keyword/Abstract = Chinese-English expanded search (acupuncture therapy) |
| 6 | Title/Keyword/Abstract = Chinese-English expanded search (acupuncture treatment) |
| 7 | OR/1–6 |
| 8 | Title/Keyword/Abstract = Chinese-English expanded search (migraine) |
| 9 | Title/Keyword/Abstract = Chinese-English expanded search (brain imaging) |
| 10 | Title/Keyword/Abstract = Chinese-English expanded search (neuroimaging) |
| 11 | Title/Keyword/Abstract = Chinese-English expanded search (magnetic resonance imaging) |
| 12 | Title/Keyword/Abstract = Chinese-English expanded search (nuclear magnetic resonance) |
| 13 | Title/Keyword/Abstract = Chinese-English expanded search (functional magnetic resonance imaging) |
| 14 | Title/Keyword/Abstract = Chinese-English expanded search (fMRI) |
| 15 | Title/Keyword/Abstract = Chinese-English expanded search (regional homogeneity) |
| 16 | Title/Keyword/Abstract = Chinese-English expanded search (ReHo) |
| 17 | Title/Keyword/Abstract = Chinese-English expanded search (amplitude of low-frequency fluctuation) |
| 18 | Title/Keyword/Abstract = Chinese-English expanded search (ALFF) |
| 19 | Title/Keyword/Abstract = Chinese-English expanded search (positron emission tomography) |
| 20 | Title/Keyword/Abstract = Chinese-English expanded search (PET) |
| 21 | Title/Keyword/Abstract = Chinese-English expanded search (single-photon emission computed tomography) |
| 22 | Title/Keyword/Abstract = Chinese-English expanded search (SPECT) |
| 23 | Title/Keyword/Abstract = Chinese-English expanded search (cerebral blood flow) |
| 24 | Title/Keyword/Abstract = Chinese-English expanded search (CBF) |
| 25 | Title/Keyword/Abstract = Chinese-English expanded search (arterial spin labeling) |
| 26 | Title/Keyword/Abstract = Chinese-English expanded search (ASL) |
| 27 | OR/9–26 |
| 28 | 7 AND 8 AND 27 |
| **CBM** | |
| 1 | Acupuncture Therapy [unweighted exploded] |
| 2 | needle OR acupuncture OR acupuncture needling OR electroacupuncture OR acupuncture therapy OR acupuncture treatment [common fields: intelligent] |
| 3 | Migraine [unweighted exploded] |
| 4 | migraine [common fields: intelligent] |
| 5 | Magnetic Resonance Imaging [unweighted exploded] |
| 6 | brain imaging OR neuroimaging OR magnetic resonance imaging OR nuclear magnetic resonance OR functional magnetic resonance imaging OR amplitude of low-frequency fluctuation OR ALFF OR positron emission tomography OR PET OR single-photon emission computed tomography OR SPECT OR cerebral blood flow OR brain perfusion OR CBF OR arterial spin labeling OR ASL [common fields: intelligent] |
| 7 | 1 OR 2 |
| 8 | 3 OR 4 |
| 9 | 5 OR 6 |
| 10 | 7 AND 8 AND 9 |
| **Wanfang** | |
| 1 | Topic: acupuncture therapy |
| 2 | Title or Keyword: needle OR acupuncture OR acupuncture needling OR electroacupuncture OR acupuncture therapy OR acupuncture treatment |
| 3 | 1 OR 2 |
| 4 | Topic: migraine |
| 5 | Title or Keyword: migraine |
| 6 | 4 OR 5 |
| 7 | Topic: magnetic resonance imaging |
| 8 | Title or Keyword: brain imaging OR neuroimaging OR magnetic resonance imaging OR nuclear magnetic resonance OR functional magnetic resonance imaging OR fMRI OR regional homogeneity OR ReHo OR amplitude of low-frequency fluctuation OR ALFF OR positron emission tomography OR PET OR single-photon emission computed tomography OR SPECT OR cerebral blood flow OR CBF OR arterial spin labeling OR ASL |
| 9 | 7 OR 8 |
| 10 | 3 AND 6 AND 9 |
| **VIP** | |
| 1 | Title or Keyword: needle OR acupuncture OR acupuncture needling OR electroacupuncture OR acupuncture therapy OR acupuncture treatment |
| 2 | Title or Keyword: migraine |
| 3 | Title or Keyword: brain imaging OR neuroimaging OR magnetic resonance imaging OR nuclear magnetic resonance OR functional magnetic resonance imaging OR fMRI OR regional homogeneity OR ReHo OR amplitude of low-frequency fluctuation OR ALFF OR positron emission tomography OR PET OR single-photon emission computed tomography OR SPECT OR cerebral blood flow OR CBF OR arterial spin labeling OR ASL |
| 4 | 1 AND 2 AND 3 |

**fMRI, functional magnetic resonance imaging; ReHo, regional homogeneity; ALFF, amplitude of low-frequency fluctuation; SPECT, single-photon emission computed tomography;** PET, positron emission tomography; ASL, arterial spin labeling; MeSH, Medical Subject Headings; CBF, cerebral blood flow.

Supplementary Table 2. Quality assessment checklist.

| # | Category | Criterion |
| --- | --- | --- |
| 1 | Sample characteristics | Patients were prospectively evaluated using explicit diagnostic criteria, and demographic data such as age and sex were reported. |
| 2 | Sample characteristics | Key clinical variables, including disease duration, attack frequency, VAS scores, medication status, comorbidities, or illness severity, were documented and controlled through statistical methods or stratification. |
| 3 | Sample characteristics | Acupuncture procedures, including acupoint selection, needling methods, and treatment course, were clearly described. |
| 4 | Sample characteristics | Each study group included more than ten participants, and the handling of dropouts or missing data was reported. |
| 5 | Image acquisition and analysis | MRI was performed with a field strength of at least 1.5 T. |
| 6 | Image acquisition and analysis | Resting-state acquisition lasted longer than five minutes. |
| 7 | Image acquisition and analysis | Whole-brain automated analysis was conducted without the use of predefined regions of interest. |
| 8 | Image acquisition and analysis | Spatial coordinates were reported in a standard stereotactic space such as MNI or Talairach. |
| 9 | Image acquisition and analysis | Image preprocessing and statistical analysis procedures, including denoising, smoothing, normalization, or filtering, were described in sufficient detail to ensure reproducibility. |
| 10 | Image acquisition and analysis | Appropriate methods for multiple-comparison correction were applied, including FWE, FDR, or permutation-based approaches. |
| 11 | Results and conclusions | Statistical parameters, including significant and non-significant findings, were reported, along with peak coordinates and cluster sizes. |
| 12 | Results and conclusions | The conclusions were consistent with the results and included discussion of study limitations and clinical relevance. |

Note: Each criterion was scored as 1, 0.5, or 0, indicating that the criterion was fully met, partially met, or not met, respectively. A score of 0.5 was assigned when the relevant information was reported but incomplete or when the criterion was only partially satisfied. Total scores ranged from 0 to 12. Studies with total scores >10, 6–10, and <6 were classified as high, moderate, and low quality, respectively.

VAS, Visual Analogue Scale; MRI, **magnetic resonance imaging;** MNI, Montreal Neurological Institute; FWE, family-wise error; FDR, false discovery rate.

Supplementary Table 3. Item level quality assessment scores for the 13 included studies.

|  |  | Category 1: Samples | | | | Category 2: Methods for image acquisition and analysis | | | | | | Category 3: Results and conclusions | |  | Tips：Score (0/0.5/1) when criteria were partially met, 0.5 points were assigned |
| --- | --- | --- | --- | --- | --- | --- | --- | --- | --- | --- | --- | --- | --- | --- | --- |
| Study ID | Name & Year | Clear diagnostic criteria; age and gender reported | Key clinical variables documented and controlled | Acupuncture procedures clearly described | >10 per group; dropouts reported | MRI ≥1.5T | Resting-state scan >5 minutes | Whole-brain automated analysis | Coordinates in standard space (MNI/Talairach) | Preprocessing procedures described | Multiple comparison correction applied | Statistical parameters and coordinates reported | Conclusions match results; limitations discussed | Total |  |
| 1 | Gaoxiaoyu 2025 | 1 | 1 | 1 | 1 | 1 | 1 | 1 | 1 | 1 | 1 | 1 | 1 | 12 |  |
| 2 | Hanxiao 2017 | 0 | 0 | 1 | 1 | 1 | 1 | 1 | 1 | 1 | 1 | 1 | 1 | 10 |  |
| 3 | Ningyanzhe 2020 | 1 | 0.5 | 1 | 1 | 1 | 1 | 1 | 1 | 1 | 1 | 1 | 1 | 11.5 |  |
| 4 | Jiajingnan 2021 | 1 | 0.5 | 1 | 1 | 1 | 1 | 1 | 1 | 1 | 1 | 1 | 1 | 11.5 |  |
| 5 | Wangziwen 2021 | 0 | 0.5 | 1 | 0.5 | 1 | 1 | 1 | 1 | 1 | 1 | 1 | 1 | 10 |  |
| 6 | Zhangyanan 2021 | 1 | 1 | 1 | 1 | 1 | 1 | 1 | 1 | 1 | 1 | 1 | 1 | 12 |  |
| 7 | Zhaoling 2014 | 1 | 1 | 1 | 1 | 1 | 1 | 1 | 1 | 1 | 1 | 1 | 1 | 12 |  |
| 8 | Zhangyutong 2021 | 0.5 | 1 | 1 | 1 | 1 | 1 | 1 | 1 | 1 | 1 | 1 | 1 | 11.5 |  |
| 9 | Yangyuchan 2024 | 1 | 1 | 1 | 1 | 1 | 1 | 1 | 1 | 1 | 1 | 1 | 1 | 12 |  |
| 10 | Xiechaorong 2021 | 1 | 1 | 1 | 1 | 1 | 1 | 1 | 1 | 1 | 1 | 1 | 1 | 12 |  |
| 11 | Lizhengjie 2017 | 1 | 1 | 1 | 1 | 1 | 1 | 1 | 1 | 1 | 1 | 1 | 1 | 12 |  |
| 12 | Liushanshan 2021 | 1 | 1 | 1 | 1 | 1 | 1 | 1 | 1 | 1 | 1 | 1 | 1 | 12 |  |
| 13 | Lichong 2023 | 1 | 1 | 1 | 1 | 1 | 1 | 1 | 1 | 0 | 0.5 | 1 | 1 | 10.5 |  |

MRI, **magnetic resonance imaging;** MNI, Montreal Neurological Institute.

Supplementary Table 4. Jackknife sensitivity analysis.

| All studies | | Right Middle Frontal Gyrus | Left Angular Gyrus | Right Superior Temporal Gyrus | Right Precuneus |
| --- | --- | --- | --- | --- | --- |
| ReHo | | | | | |
|  | Gao *et al*. [38] 2025 |  | √ |  |  |
|  | Han *et al*. [39] 2017 |  | √ | √ | √ |
|  | Jia *et al*. [41] 2021 |  | √ | √ | √ |
|  | Wang *et al*. [42] 2021a |  | √ | √ | √ |
|  | Wang *et al*. [42] 2021b |  | √ | √ |  |
|  | Zhang *et al*. [43] 2024 |  | √ | √ | √ |
|  | Zhao *et al*. [44] 2014 |  |  |  |  |
|  | Zhang *et al*. [45] 2021 |  | √ |  | √ |
|  | Xie *et al*. [47] 2024 |  | √ | √ |  |
|  | Liu *et al*. [49] 2021a |  | √ | √ | √ |
|  | Liu *et al*. [49] 2021b |  | √ |  |  |
|  | Li *et al*. [50] 2023 |  | √ | √ | √ |
| ALFF | | | | | |
|  | Ning *et al*. [40] 2020 |  |  |  |  |
|  | Zhang *et al*. [45] 2021 |  |  |  |  |
|  | Yang *et al.* [46] 2024 | √ |  |  |  |
|  | Xie *et al*. [47] 2024a |  |  |  |  |
|  | Xie *et al*. [47] 2024b | √ |  |  |  |
|  | Li *et al*. [48] 2017 | √ |  |  |  |
|  | Li *et al*. [50] 2023 | √ |  |  |  |
| Total | | 4 out of 7 | 11 out of 12 | 8 out of 12 | 7 out of 12 |

Letter suffixes indicate different analyses from the same study: Wang *et al*. [42] 2021 (a = local acupoints, b = distal acupoints); Xie *et al*. [47] 2024 (a = ALFF, b = fALFF); Liu *et al*. [49] 2021 (a = immediate effects, b = cumulative effects).

**ReHo, regional homogeneity; ALFF, amplitude of low-frequency fluctuation.**

**Supplementary Fig. 1. Funnel plots of peak MNI coordinates.** (A) Peak MNI coordinates of clusters with reduced ALFF after acupuncture. (B–E) Peak MNI coordinates of clusters with reduced and increased ReHo after acupuncture.
